# Are Parents Patient? The Influence of Parenting Role Salience and Parental Status on Impatience

**DOI:** 10.3389/fpsyg.2018.01523

**Published:** 2018-08-22

**Authors:** Yuanyuan J. Li, Yuanyuan Liu

**Affiliations:** ^1^Department of Marketing and E-Business, School of Business, Nanjing University, Nanjing, China; ^2^Department of Marketing, Xi’an Jiaotong University, Xi’an, China

**Keywords:** parent vs. non-parent, parental role salience, intertemporal discounting task, impatience, risk aversion, resource accumulation

## Abstract

In the classic intertemporal discounting task (Thaler, 1981), individuals make tradeoff decisions between smaller-sooner and larger-later monetary rewards. We explored how parental role salience and parental status influences individual’s choice between smaller-sooner and larger-later choices. Parental role salience is manipulated among both parents and non-parents in this research. Our results show a significant interaction between parental status and manipulated parental role salience. Specifically, we found that parents are more impatient than non-parents. Additionally, non-parents become more impatient after parental role salience manipulation, similar to parents. Theoretical implications of our findings are discussed.

## Introduction

Individuals often make tradeoffs between the magnitude and the delivery timing of an outcome, in another word, between a smaller-sooner and a larger-later outcome. Delayed rewards have less value than immediate ones, and individuals are willing to sacrifice some amount of rewards for the immediacy, which is widely known as impatience. Earlier research shows that individuals are impatient and future rewards are often discounted heavily in such tradeoffs (e.g., Frederick et al., 2002).

Several sources may contribute to the discounting of delayed rewards (see Hardisty et al., 2013). First, decision-makers prefer instant gratification and thus find delays frustrating (O’Donoghue and Rabin, 1999). Also, imminent rewards may activate the brain’s reward system and lead to impulsivity (Loewenstein, 1996). Second, the future is inherently associated with risk, because we have less information and control over the future (Mischel et al., 2003; Patak and Reynolds, 2007), such that future payoffs may not be realized due to natural disasters, uncertain lifetime, and the credibility of the promise. Halevy (2008) also argues that the central difference between the present and the future is the certainty of the former and the uncertainty of the latter. Other factors also contribute to future discounting, including opportunity cost consideration (Zhao et al., 2015) and the resource slack hypothesis that people may misperceive their resource abundance in the future than in the present time (Zauberman and Lynch, 2005). Existing research shows that future discounting may be influenced by various factors, including visceral factors (Loewenstein, 1996), certainty of the outcomes (Keren and Roelofsma, 1995; Weber and Chapman, 2005), monetary priming (Jiang et al., 2016), power (Duan et al., 2017), and mental representation (Malkoc et al., 2010). In the current paper, we are interested in the influence of parental role salience on intertemporal choices.

Parenting is one of the most important, essential, and difficult things in human evolution (Buckels et al., 2015). Human babies are born extremely vulnerable, and are the costliest to rear among all primate infants (Cárdenas et al., 2013; Preston, 2013). The average cost for parents to raise up one single child in the United States is estimated to be between $205,960 and $475,680, without considering college tuition (Lino, 2010). Due to the importance of parenting in evolution, individuals who exhibit traits and psychological tendencies consistent with parental roles ensure a higher survival rate of dependent offspring, thus are adaptive (Preston, 2013). Moreover, a specific role could be primed to be temporarily salient, like parental role. Under parental role priming, individuals exhibit traits and pursue goals in line with those roles (Fitzsimons and Bargh, 2003). Therefore, it is an important question to explore how parental role salience influences individual’s behavior in economic decisions, like intertemporal choice.

Prior literature makes conflicting predictions on how parental role salience influences intertemporal choice. One stream of literature argues that parenthood, like parental role, increases financial resource need and therefore enhances the focus on reward’s quantity in intertemporal choices (Solomon and George, 1996). Based on this line of research, we may expect that parental role salience increases willingness to wait for larger-later rewards (Nenkov and Scott, 2014). Another stream of literature predicts the opposite. That is, delaying outcomes naturally entails risks (Keren and Roelofsma, 1995; Weber and Chapman, 2005). The longer the delay, the less likely individuals are to receive the outcome in the future. Therefore, being impatient could be an efficient way to avoid risks because immediate outcomes are more certain than delayed ones (Weber and Chapman, 2005; Kidd et al., 2013). Previous literature shows both correlational and causal relationships between parental role salience and risk-vigilant perceptions (Solomon and George, 1996; Wang et al., 2009; Cameron et al., 2010; Eibach and Mock, 2011). Specifically, parental role salience increases risk perception and risk-averse choices among parents (Eibach and Mock, 2011). Since risk avoidance is one important contributor of impatience (Bommier, 2006), we expect that parental role salience may increase impatience in intertemporal choices.

In the present experiment, we measured parental status and manipulated parental role salience. Specifically, an experiment was conducted to compare whether parents are financially more impatient than non-parents; whether manipulated parental role salience increases financial impatience; whether manipulated parental role salience has a stronger effect among parents or non-parents.

## Materials and Methods

### Participants

Two hundred and eighty three participants (*M*_age_ = 36.54, *SD*_age_ = 11.38; man = 159) were recruited online from Amazon mechanical turk, and the experiment was programmed on *Qualtrics*. All subjects received $0.19 as rewards.

### Experiment Design and Procedure

This was a 2 (parental status: parent vs. non-parent) × 2 (manipulation: parental role salience vs. not salience) between subjects design with parental status measured and parental role salience manipulated by changing the order of parenthood-related questions (Eibach et al., 2009; Eibach and Mock, 2011). In parental role salience conditions, the discounting task was placed after parenthood-related questions, which were: (1) “are you parents,” (2) “how many children do you have,” and (3) “how old is your youngest child.” In control conditions, the discounting task was placed before the parenthood-related questions. Previous literature has shown that participants who reported their parenthood-related questions before the dependent variable had a higher parental role salience as compared with those who reported parenthood-related questions after the dependent variable (Eibach et al., 2009; Eibach and Mock, 2011).

Impatience was measured by the classic delay-discounting task (Thaler, 1981). Subjects were asked to specify the amount of money they prefer to receive between serials of a smaller-sooner and a larger-later monetary option, until the indifferent value was reached (e.g., “I am indifferent between receiving $__ now vs. receiving $150 in one week”). The initial smaller-sooner option was set to be $75 (**Supplementary Figure S1**). If subjects preferred the larger-later option in the first round, the smaller-sooner option increased to be $112.5; if subjects preferred the smaller-sooner in the first round, the smaller-sooner option decreased to be $37.5. Three time points were included: one week, two weeks, and one month. Serials of repeated choices were made until the indifferent values between receiving $X now and receiving $150 in one week, in two weeks, and in one month are reached. Following Myerson et al. (2001), these indifference values were then used to draw a two-dimensional discounting curve for each participant with time points on the X-axis and subjective (indifference) values on the Y-axis, and discounting was measured by the area under the discounting curve. The area under the discounting curve ranges from 0 (steepest possible discounting and extreme impatience) to 1 (no discounting at all and extreme patience).

## Results

Following Myerson et al. (2001), the area under the discounting curve (ranging from 0 to 1) measures the level of impatience. A smaller score (area under the discounting curve) indicates a higher level of impatience. Due to the non-normal distribution of the dependent variable (skewness = -0.76, Kurtosis = -0.70), we composed a new dependent variable: first, the initial dependent variable was reversed (1-area), then log-transformed, thus the higher score in the new dependent variable indicate more impatience (**Table 1**).

**Table 1:** The average indifferent smaller-sooner values of $150 at diverse time slots across conditions.

	Parental status	N	One week	Two weeks	One month	Area under the discounting curve	Log-transform (higher score, more impatience)
							
			Mean	*SD*	Mean	*SD*	Mean	*SD*	Mean	*SD*	Mean	*SD*
Role salience manipulation	Parent	90	107.53	45.41	103.72	47.23	102.01	44.97	0.73	0.25	−2.10	1.58
	Non-parent	52	122.00	43.01	114.69	39.69	103.25	43.43	0.78	0.23	−2.18	1.58

Control	Parent	96	106.91	42.52	103.54	43.21	87.48	50.23	0.70	0.24	−1.79	1.28
	Non-parent	45	128.43	34.95	123.40	36.86	119.00	40.38	0.84	0.22	−2.95	1.59

We conducted a stepwise regression to explore how parental role salience influences financial impatience. Because parental role salience manipulation and parental status were categorical variables, both variables were coded as dummy variables. In the salience manipulation, “1” denoted salience, and “0” denoted not salience. For parental status, “1” denoted parent, and “0” denoted non-parent. Due to the high correlation between age and parental status (*r* = 0.24^∗∗^), age (centered) entered the regression model first. Then, parental role salience manipulation and parental status entered in the second regression model. Third, model 3 included the interaction between parental role salience manipulation and parental status. Results revealed that parents were more financially impatient than non-parents, as they had a smaller area under the discounting curve. Parental role salience manipulation did not have a significant effect on impatience. Moreover, the interaction between parental role salience manipulation and parental status was significant (**Table 2**). Spotlight analysis revealed that manipulated parental role salience increased financial impatience among non-parents, but not among parents which may be due to a ceiling effect because parents were already at a high level of impatience.

**Table 2:** The influence of parental role salience and parental status on intertemporal choice.

	(1)	(2)	(3)
Age	−0.01 (0.01)	−0.02 (0.01)^∗^	−0.02 (0.01)^∗^
Parental role salience		0.09 (0.18)	0.79 (0.29)
Parental status		0.70 (0.19)^∗∗∗^	1.26 (0.27)^∗∗∗^
Parental role salience × Parental status			−1.07 (0.36)^∗∗^
R square adjusted	0.01	0.047	0.029

*Dependent variable: Log-transform (1-Area). ^∗^p < 0.05, ^∗∗^p < 0.01, ^∗∗∗^p < 0.001.*

Alternatively, due to the non-normal distribution of the dependent variable (area under the discounting curve), a nonparametric test could be applied as well. Mann–Whitney test revealed consistent findings as the regression analyzes above. In general, parents were more impatient than non-parents (*z* = -3.36, *p* = 0.001), while manipulated parental role salience had no impact on subjects’ choices in the intertemporal discounting task (*z* = -0.09, *p* = 0.93). Moreover, separate Mann–Whitney test by parental status shows that parental role salience increased financial impatience only among non-parents (*z* = -2.43, *p* = 0.02), not among parents (*z* = -1.06, *p* = 0.29). Similarly, in the baseline, parents had a significantly higher preference for smaller-sooner over larger-later options than non-parents (*z* = -3.87, *p* < 0.01). However, when the parental role was manipulated salience, the difference between parents and non-parents disappeared (*z* = -0.83, *p* = 0.41).

## Discussion

This study aims at testing how parental role salience influences impatience. We manipulated parental role salience by placing the parenthood-related questions either before or after the intertemporal discounting task. Meanwhile, participants’ actual parental status was measured. Our results show a significant main effect of parental status, such that parents are more impatient than non-parents. Moreover, consistent with our predictions, manipulated parental role salience interacts with parental status on intertemporal discounting task. For parents, manipulated parental role salience does not have a significant effect on individual’s intertemporal choices. On the contrary, manipulated parental role salience drives non-parents to be more impatience, acting like a parent.

Our findings might be driven by the relationship between parenthood and risk-aversion (Eibach and Mock, 2011). In the intertemporal discounting task, participants have to make tradeoffs between smaller-sooner and larger-latter monetary rewards (Thaler, 1981). The tradeoff between the magnitude of rewards and delivery timing of rewards may be driven by different processes (Mischel et al., 2003; Weber and Chapman, 2005). Our results suggest that the rewards’ delivery timing overrides the magnitude when the parental role becomes salient. Although parenting may increase the need for resources (Cárdenas et al., 2013; Preston, 2013), our finding is more consistent with the risk aversion account that parenting increased risk perception and risk-averse choices among parents (Eibach and Mock, 2011). Specifically, parenting may prioritize the delivery of rewards over quantity of rewards, which drives impatience in the intertemporal discounting task.

Though in some research, such as the classic Marshmallow experiment, delaying gratification is defined as self-control (Mischel and Ebbesen, 1970; Mischel and Baker, 1975), and impatience is often considered as one manifestation of self-regulation failure, our results that parents are less patient does not necessarily imply parenthood increases self-regulation failure. Because waiting for larger-later rewards may end up with nothing, therefore, trading an uncertain larger-latter outcome for a sure smaller-sooner outcome could be rational and adaptive (Mischel et al., 2003; Kidd et al., 2013). In the tradeoff between payoffs size and delivery time, parenthood adds the weight of probability to ensure the option delivery, thus becomes more impatient.

The present study has two major limitations. First, although there is no strong evidence to predict an interfere by actual payoff, our findings will be more convincing if real payoffs were provided in our study. Second, parental role salience manipulation did not have a significant influence on parental status in our study. We speculate that this indifference may be driven by a ceiling effect. Further studies are needed to explore whether parental role salience could be strengthened among parents.

## Conclusion

Our study investigates how parental role salience influences impatience in the intertemporal discounting task. Parents are financially more impatient than non-parents. Although manipulated parental role salience did not have a main effect on impatience, the significant interaction between parental status and parental role salience shows that manipulated parental role salience only increases financial impatience among non-parents. This finding is consistent with and extends the stream of literature about role priming that non-parents could act like a parent by parental role salience manipulation.

## Ethics Statement

This study was carried out in accordance with the recommendations of APA’s ethical guidelines, Research Ethical Committee of Business School, Nanjing University. The protocol was approved by the Research Ethical Committee of Business School, Nanjing University. All subjects gave informed consent prior to participation in the study. All subjects could abort the experiment at any time.

## Author Contributions

YJL contributed to the conception of the work, data acquisition, analysis, and interpretation, as well as drafting and revising the manuscript. YL contributed to the conception of the work, data interpretation, as well as revising the manuscript. Both authors approved the final version.

## Conflict of Interest Statement

The authors declare that the research was conducted in the absence of any commercial or financial relationships that could be construed as a potential conflict of interest.

## Supplementary Material

FIGURE S1 Initial setting of smaller-sooner option is $75. **(B)** If first choosing larger-later option, the smaller-sooner option becomes $112.5. **(C)** If first choosing smaller-sooner option, the smaller-sooner option becomes $37.5.
