# Supplementary figures and images for: Are Parents Patient? The Influence of Parenting Role Salience and Parental Status on Impatience
Source: Front Psychol. 2018 Aug 22;9:1523. doi: 10.3389/fpsyg.2018.01523 (PMC6113944; doi:10.3389/fpsyg.2018.01523)

**Supplementary Materials:**

Figure 1a


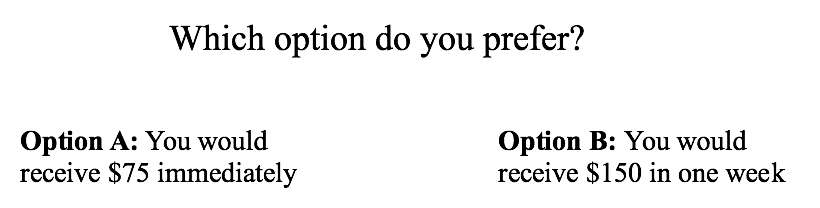


Figure 1b


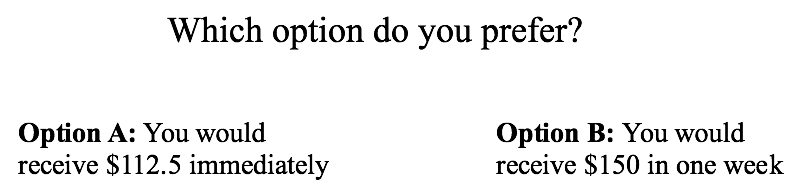


Figure 1c


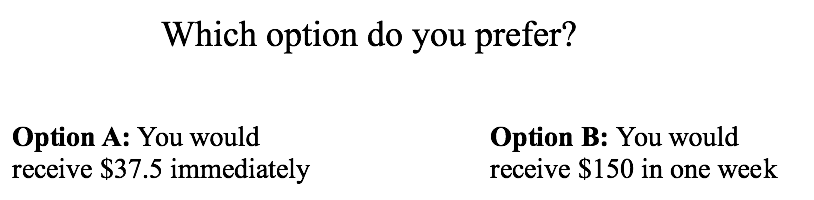

Supplement: FIGURE S1 — Initial setting of smaller-sooner option is $75. (B) If first choosing larger-later option, the smaller-sooner option becomes $112.5. (C) If first choosing smaller-sooner option, the smaller-sooner option becomes $37.5. [file Table_1.DOCX]
